# Menstrual irregularity and bone mass in premenopausal women: Cross-sectional associations with testosterone and SHBG

**DOI:** 10.1186/1471-2474-11-288

**Published:** 2010-12-29

**Authors:** Shuying Wei, Graeme Jones, Russell Thomson, Petr Otahal, Terry Dwyer, Alison Venn

**Affiliations:** 1 Menzies Research Institute, University of Tasmania, Hobart, Australia; 2 Murdoch Children's Research Institute, Victoria, Australia

## Abstract

**Background:**

There have been few studies examining the associations between menstrual irregularity, androgens and bone mass in population-based samples of premenopausal women. This study aimed to describe the associations between menstrual pattern, testosterone, sex hormone binding globulin (SHBG) and bone mass in a population-based sample of premenopausal women.

**Methods:**

Cross-sectional study (N = 382, mean age 31.5 years). Menstrual pattern was assessed by questionnaire, bone mass measured by quantitative ultrasound (QUS) and androgen status was assessed by levels of serum testosterone, SHBG and the free androgen index (FAI).

**Results:**

Women with irregular cycles (n = 41, 11%) had higher free androgen index (FAI, P = 0.01) and higher QUS measurements including speed of sound (SOS, 1%, P < 0.05), quantitative ultrasound index (QUI, 7%, p < 0.05), and broadband ultrasound attenuation (BUA, 7%, p = 0.10). These associations persisted after adjustment for age and body mass index (BMI). After further adjustment for hormonal factors (either testosterone, SHBG or FAI), the strength of the associations was moderately attenuated, however, women with irregular cycles still had a 6% increase in mean QUS. Total testosterone, FAI and SHBG were also associated with QUS measures (testosterone and FAI, r +0.11 to +0.21, all p < 0.05; SHBG r -0.14 to -0.16, all p < 0.05) and the associations remained significant after adjustment.

**Conclusion:**

Irregular menstrual cycles were associated with higher bone mass in this population-based sample of premenopausal women suggesting menstrual disturbance should continue to be evaluated but may be less harmful for bone mass. The association between menstrual irregularity and bone mass was partially mediated by markers of androgen status especially free testosterone.

## Background

Osteoporosis is a major public health concern [1]. Most osteoporosis sufferers are postmenopausal women and age-related estrogen deficiency is considered a major cause of bone loss [2]. As well as estrogen, androgens may also have an effect on bone metabolism in women; excess levels of androgen are associated with higher bone mass in women with Polycystic Ovary Syndrome (PCOS) [3-7]. Menstrual irregularity has been associated with lower bone mass in studies of female athletes who had low BMI and extensive training [8-11]. However these results may not directly apply to today's population of young women with a higher prevalence of overweight and obesity. Recent studies have suggested that menstrual irregularity is associated with higher levels of androgens in a population-based sample of premenopausal women [12,13], while in female elite athletes oligomenorrhea was associated with higher bone mass and PCOS [14]. These results may in turn suggest a positive association between menstrual irregularity and bone mass. However, few studies have examined the associations between menstrual irregularity, androgen and bone mass in population-based sample. Therefore, the aim of this cross-sectional study was to describe the association between menstrual pattern, testosterone, sex hormone binding globulin (SHBG) and bone mass measured by quantitative ultrasound (QUS) in a population-based sample of young women.

## Methods

### Subjects

This study utilized data from the Childhood Determinants of Adult Health (CDAH) study, a 20-year follow-up of children who were randomly selected for the 1985 Australian Schools Health and Fitness Survey (ASHFS) at age 7-15 years. Details of the 1985 sampling strategy have been described elsewhere [15]. During follow-up (2004-2006) a total of 2410 subjects aged 26-36 years (48% male, 52% female), completed questionnaires and attended one of 34 study clinics in major cities and regional centres around Australia for extensive physical measurements including anthropometric measures, quantitative ultrasound and blood biochemistry.

Eligible subjects for this study were women who had completed study questionnaires, attended study clinics, were not currently taking oral contraceptives, and not pregnant or breast feeding at the time of clinic attendance. Of the 1260 women who attended clinics, 82 were currently pregnant; 453 were currently taking hormonal contraceptives (including combined and progestin only contraceptives and progestin releasing intrauterine devices) or medications such as hormonal agents for PCOS or in vitro fertilization (IVF); 44 did not provide their menstrual cycle characteristics; and 299 had missing QUS measurements due to the machine being out of service, leaving a total of 382 participants for this analysis. All participants provided written informed consent and the study was approved by the Southern Tasmania Health and Medical Human Research Ethics Committee.

### Assessment of menstrual cycle characteristics

Menstrual cycle characteristics were obtained by written questionnaire. We defined the menstrual cycle as the time from the first day of one period to the first day of the next. The question was then asked: " thinking about the most recent time when you were having periods and were not using hormonal contraceptives (e.g., the pill) and were not pregnant or breast feeding: would you describe your period as very regular, fairly regular, irregular or very irregular [16]. To simplify analyses, menstrual cycle pattern was defined as regular if women described their periods as very or fairly regular, and irregular if irregular or very irregular.

### Hormone measurements

Blood samples (32 ml) were collected from participants after an overnight fast. Plasma insulin was initially measured by a microparticle enzyme immunoassay kit (AxSYM; Abbot Laboratories, Abbot Park, IL) and later, following a change in the choice of kit by the testing laboratory, by electrochemiluminescence immunoassay (Elecsys Modular Analytics E170; Roche Diagnostics, Mannheim, Switzerland). Due to this change in assay methodology, insulin levels from participants' samples (N = 258) assayed using the first methodology were corrected to levels in samples assayed using the second methodology (as per correction factor equation of the laboratory). Total testosterone concentrations were estimated by radioimmunoassay (RIA) developed by Repromed Laboratory (Dulwich, South Australia), which is sensitive for lower levels of testosterone down to 347 pmol/L. SHBG was measured using a non-competitive liquid-phase immunoradiometric assay (SHBG-IRMA kit, Orion Diagnostica, Espoo, Finland). For testosterone, the intra- and inter- assay coefficients of variation (CV) were 6% at 1 nmol/l and 15%, respectively. For SHBG, the intra- and inter- assay CV were 15.4%, and 2.0-8.6% respectively. FAI was calculated as: testosterone (nmol/L) * 100/SHBG (nmol/L).

### Anthropometric measurements

Anthropometric measures were taken at study clinics by trained personnel. Height and weight were measured to the nearest 0.1 cm and 0.1 kg, respectively, and body mass index (BMI) was calculated as the ratio of weight (kg) to height (m) squared (kg/m^2^). Waist to hip ratio (WHR) was calculated by dividing waist by hip circumference measured to the nearest 0.1 cm.

### Quantitative ultrasound measurements

All participants had calcaneal QUS measured by a single Sahara Clinical Bone Sonometer (Hologic Inc., MA, USA). The ultrasound system consists of two sound transducers mounted coaxially on a motorized calliper with one transducer acts as an emitter and the other as a receiver. This makes direct contact with the heel through elastomer pads and an ultrasonic coupling gel. The lower part of the dominant leg was placed immobilized during measurement and the proper leg angle set by a positioning aid. Broadband ultrasound attenuation (BUA, dB/MHz) and speed of sound (SOS, m/s) were measured at a fixed region in the mid-calcaneus. The quantitative ultrasound index (QUI) was derived from SOS and BUA using the equation: QUI = 0.41× (BUA+SOS)-571 [17]. Quality assurance was performed daily by calibrating the device on a dedicated phantom supplied by the manufacturer. The coefficient of variation (CV) for QUS measures was 1%.

### Assessment of other covariates

Demographic and lifestyle information was obtained by questionnaire. Smoking status was classified as non-smoker or current smoker. Women were classified as nulliparous if they had not had a live birth or parous if they had had at least one live birth. Physical activity was assessed as we have described previously [18] by the International Physical Activity Questionnaire (IPAQ) and as mean steps per day measured by Yamaz pedometer worn for seven days. The questionnaire asked whether participants had ever been told by a doctor that they had polycystic ovaries (PCO) or polycystic ovary syndrome (PCOS).

### Statistical Analysis

The unequal variance t-test was used to assess differences in normally-distributed continuous characteristics in this study across categorized menstrual cycle pattern (regular and irregular), the Wilcoxon rank-sum test was used for skewed continuous variables and the two-sample test of proportions was used for dichotomised covariates. Spearman correlations were used to estimate crude associations between continuous exposures and QUS. Multivariable linear regression analysis was used to explore the association between all QUS measures and menstrual cycle pattern and hormonal factors (testosterone, SHBG and FAI). QUS parameters and hormonal factors were logarithmically transformed to fit linear regression models and standardised to enable direct comparisons between BUA, SOS and QUI. The coefficients represent a change of one standard deviation. Age, BMI and smoking were considered as confounders or covariates of importance, and adjusted for in multivariable models where appropriate. All statistical analyses were performed on intercooled Stata 9.2 for windows (Statacorp, Texas, USA).

## Results

In this relatively young study sample with age range of 26-36 years (mean age 32 years; mean BMI 24.8 kg/m^2^), most women were married or living as married (61.7%) and were non-smokers (85.7%). Over half the women had had at least one livebirth (53.7%). Only 11% of participants had irregular menstrual cycles. Very few women sampled reported having ever been told by a doctor that they had PCO or PCOS (n = 9 with irregular cycles, n = 9 with regular cycles). Women currently taking hormonal medication for PCOS (n = 2) were ineligible for this analysis and excluded. Compared with other women in this study, women who were excluded due to current use of hormonal contraceptives or hormonal medications, were slightly younger (30.7 vs 31.5 years) and more likely to have a history of menstrual irregularity (21.6% vs 10.7%), but there was no significant difference in BMI between the groups (25.0 kg/m^2 ^vs 24.8 kg/m^2^, p = 0.54) and similar proportions were current smokers (13.2% vs 14.3%, p = 0.69).

**Figure 1:**
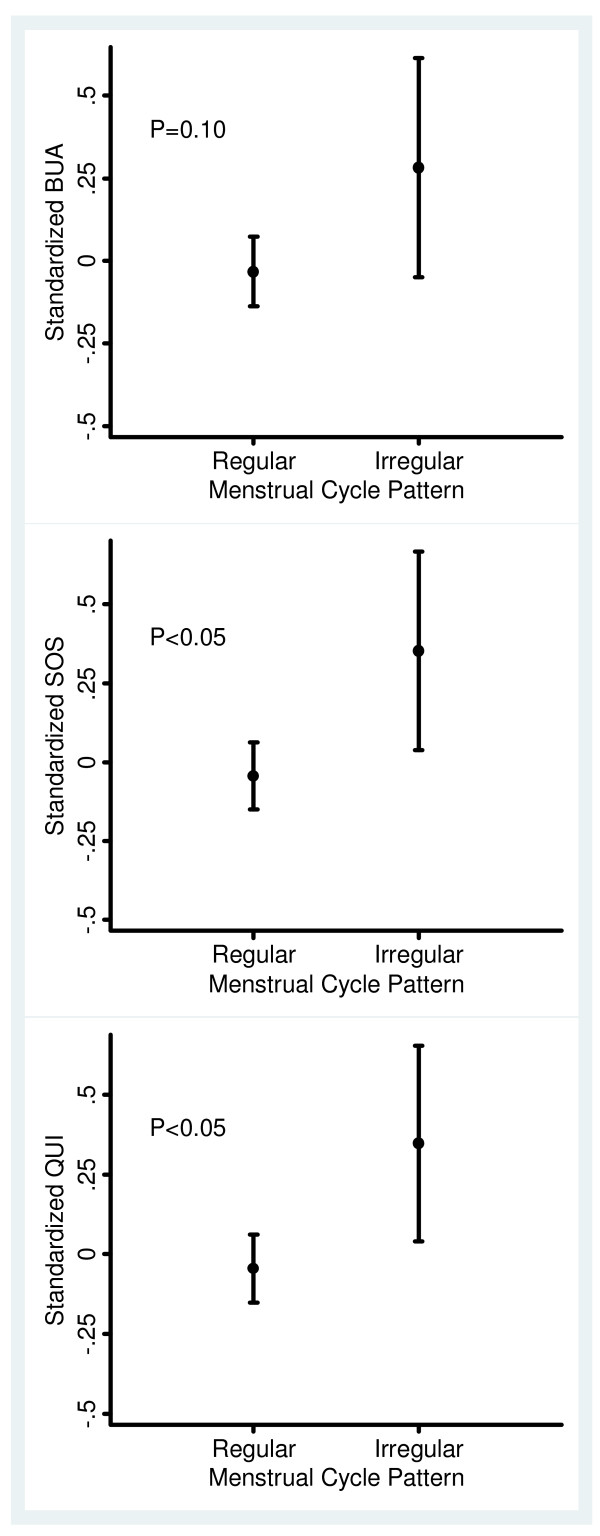
**Standardized broadband ultrasound attenuation (BUA), speed of sound (SOS) and quantitative ultrasound index (QUI) for women with regular and irregular cycles**. Error bars indicate 95% confidence intervals.

In the unadjusted analysis, women with irregular cycles had higher FAI, SOS and QUI, and lower SHBG compared with those with regular cycles (Table 1). The difference in bone mass between women with regular and irregular cycles is illustrated in Figure 1. There were no significant differences in age, body composition measurements, marital status, number of live births, smoking and plasma insulin levels between women with irregular cycles and regular cycles. However, all anthropometric measures including BMI, waist circumference and waist to hip ratio were significantly associated with BUA (p < 0.05-0.01) (Table 2) as were hormonal factors with a positive association between testosterone and FAI, and a negative association with SHBG (all p < 0.01) (data not shown).

**Table 1:** Characteristics of participants by menstrual cycle pattern

Characteristics	Regular cycle N = 341	Irregular cycle N = 41	P values
**Age (year)^a^**	31.6 (2.6)	30.9 (2.4)	0.11
**Anthropometrics ^a^**			
Body mass index (kg/m^2^)	24.8 (5.0)	24.5 (5.9)	0.77
Waist circumference (cm)	78.8 (11.4)	77.8 (13.3)	0.65
Waist-hip ratio	0.757(0.062)	0.760 (0.061)	0.75
**Married ^b ^**%	62	56	0.44
**Parous ^b ^**%	54	49	0.50
**Current smoker ^b ^**%	15	13	0.73
**Physical activity (PA) ^c^**			
Pedometer steps (per day)	8331 (6758-10623)	8325 (6715-10992)	0.68
Leisure time PA (min/week)	120 (0-219)	66 (0-180)	0.24
Total PA (min/week)	680 (385-1030)	560 (300-970)	0.27
**Hormonal factors ^c^**			
Testosterone (nmol/L)	1.50 (1.22-1.86)	1.53 (1.35-1.89)	0.24
SHBG (nmol/L)	50.3 (35.8-68.0)	43.6 (30.1-54.6)	**0.01**
Free androgen index	2.94 (2.00-4.47)	4.22 (2.60-6.24)	**0.01**
Insulin (mU/l)	3.9 (5.3-7.5)	3.5 (4.9-7.9)	0.85
**Quantitative ultrasound ^c^**:			
BUA (dB/MHz)	73.6 (65.4-84.6)	78.7 (67.9-87.1)	0.10
SOS (m/s)	1563 (1543-1587)	1569 (1557-1606)	**0.02**
QUI (%)	99.5 (88.6-114.1)	106.7 (94.6-121.6)	**0.03**

^a ^presented by mean and standard deviation (SD), P values derived from unequal variance t-test.

^b ^presented by percentage, P values derived from test of proportions. ^c ^presented by median (inter-quarter range), P values from Wilcoxon rank-sum test. Bold denotes significant results.

**Table 2:** Spearman correlation coefficients (r) for associations of quantitative ultrasound measures and continuous variables in the study

Characteristics	BUA		SOS		QUI	
	
	r	P	r	P	r	P
**Anthropometrics**						
Body mass index	**0.17**	**< 0.01**	-0.01	0.79	0.05	0.35
Waist circumference	**0.17**	**< 0.01**	-0.01	0.86	0.05	0.32
Waist-hip-ratio	**0.14**	**0.01**	0.03	0.57	0.07	0.16
**Hormonal factors**						
Testosterone	**0.14**	**0.01**	0.09	0.08	**0.11**	**0.04**
SHBG	**-0.16**	**< 0.01**	**-0.14**	**0.01**	**-0.16**	**< 0.01**
Free androgen index	**0.21**	**< 0.001**	**0.16**	**< 0.01**	**0.19**	**< 0.001**

SHBG = sex hormone-binding globulin. BUA = broadband ultrasound attenuation. SOS = speed of sound. QUI = quantitative ultrasound index. Bold denotes significant association (P < 0.05).

**Figure 2:**
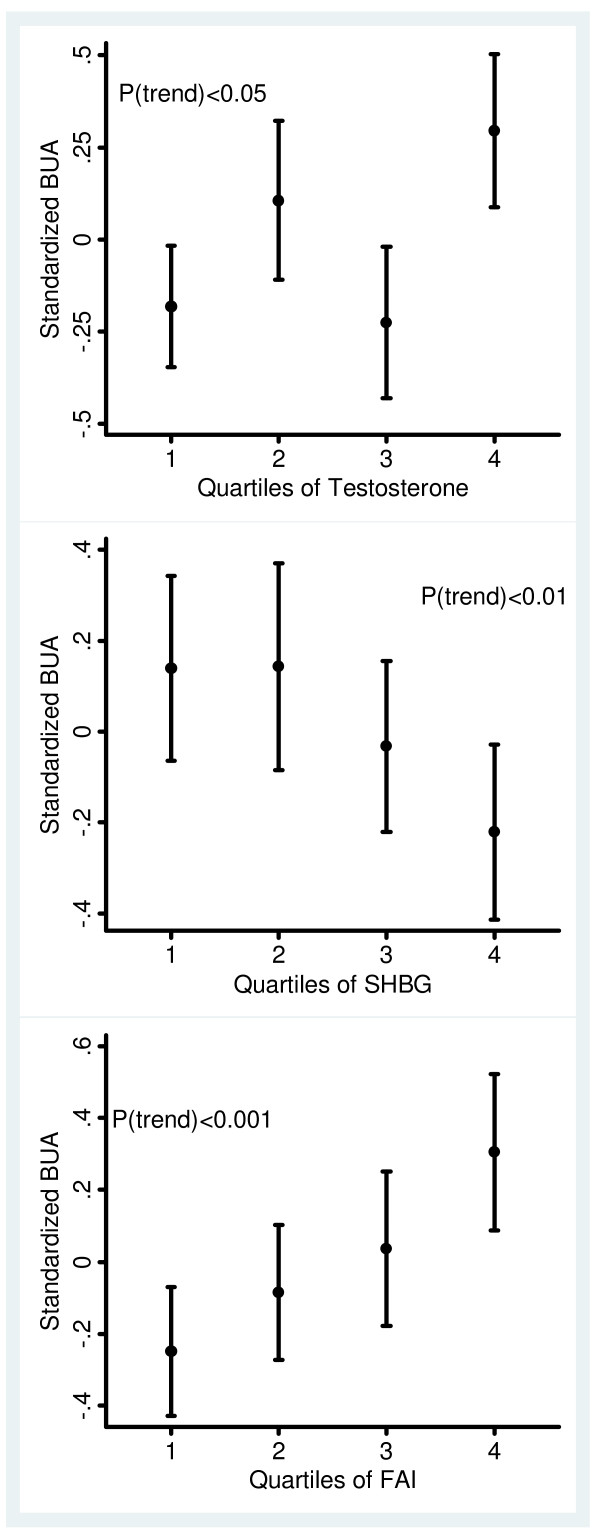
**Standardized broadband ultrasound attenuation (BUA) across quartiles of testosterone, sex hormone-binding globulin (SHBG) and free androgen index (FAI)**. Error bars indicate 95% confidence intervals. Hormonal factors were standardized parameters.

There were significant associations between hormonal factors and BUA, SOS and QUI measurements found in both unadjusted correlation analysis (Table 2) and adjusted analysis (Table 3). Total testosterone and FAI were significantly positively associated with QUS measurements, while SHBG was negatively associated with QUS measures. These associations remained significant after adjustment for confounders with the exception of SHBG and BUA. Further adjustment for history of PCO/PCOS did not change the significant associations. The association between hormonal factors and QUS measures is further illustrated in Figure 2 where there were dose-response relationships between BUA measures and hormonal factors, especially for FAI.

**Table 3:** Association between quantitative ultrasound (QUS) parameters and hormonal factors

	Unadjusted model	Adjusted model	
	
	(n = 382) β (95% CI)	Model 1 (n = 358) β (95% CI)	Model 2 (n = 358) β (95% CI)
**BUA**			
Testosterone	**0.15 (0.04, 0.24)**	**0.14 (0.04, 0.25)**	**0.13 (0.03, 0.24)**
SHBG	**-0.14 (-0.24, -0.04)**	-0.07 (-0.19, 0.05)	-0.05 (-0.17, 0.06)
FAI	**0.18 (0.08, 0.28)**	**0.14 (0.03, 0.26)**	**0.13 (0.01, 0.24)**
**SOS**			
Testosterone	**0.11 (0.01, 0.21)**	**0.13 (0.03, 0.24)**	**0.12 (0.02, 0.23)**
SHBG	**-0.13 (-0.23, -0.03)**	**-0.16 (-0.27, -0.04)**	**-0.14 (-0.26, -0.02)**
FAI	**0.16 (0.06, 0.26)**	**0.20 (0.08, 0.31)**	**0.18 (0.06, 0.29)**
**QUI**			
Testosterone	**0.12 (0.02, 0.22)**	**0.14 (0.03, 0.24)**	**0.13 (0.02, 0.23)**
SHBG	**-0.14 (-0.24, -0.04)**	**-0.13 (-0.25, -0.01)**	-0.11 (-0.23, 0.01)
FAI	**0.17(0.07, 0.27)**	**0.18 (0.07, 0.29)**	**0.16 (0.05, 0.28)**

BUA = broadband ultrasound attenuation. SOS = speed of sound. QUI = quantitative ultrasound index. SHBG = sex hormone-binding globulin. FAI = free androgen index. QUS measures and hormonal parameters were log transformed before converted into standardized variables. β = standardised beta coefficients. Model 1 adjusted for age, BMI and smoking. Model 2 further adjusted for menstrual cycle pattern. Bold denotes significant association (P < 0.05).

The associations between irregular cycles and QUS measures are presented in Table 4. In multivariable regression analysis, irregular cycles remained significant associations with SOS and QUI (β= 0.38 for both, p < 0.05), with a consistent trend for BUA (β = 0.31, p = 0.06) after adjustment for confounders. The strength of the associations was moderately attenuated when further adjusted for hormonal factors (testosterone, SHBG and FAI separately), with maximally a 27% decrease in the coefficient when further adjusted for FAI, and a lesser effect when adjusted for total levels of testosterone. However women with irregular cycles still had an increase of 0.30-0.31 in the standardized log-transformed SOS and QUI and this corresponds to a maximum 6% increase in the mean QUS measurements. We recalculated free testosterone (FT) in the equation by taking into account the laws of mass action and using a stable mean of albumin concentration (assumed to be 43 g/l), and found that the associations between menstrual irregularity and bone mass were nearly the same when adjusted for FAI and FT. Further adjustment for a history of ever been told by a doctor that they had PCO or PCOS made the associations a little stronger. When irregular and very irregular cycles were examined separately, the adjusted associations were similar in magnitude but were not statistically significant, likely due to lower power (n = 22 for irregular, n = 19 for very irregular; data not shown).

**Table 4:** Association between quantitative ultrasound (QUS) measures and menstrual irregularity adjusted by each hormonal factor separately

	Adjusted model * β (95% CI)	Adjusted model + testosterone β (95% CI)	Adjusted model + SHBG β (95% CI)	Adjusted model +FAI β (95% CI)
**BUA**				
Regular cycle	Ref	Ref	Ref	Ref
Irregular cycle	0.31 (-0.01, 0.64)	0.29 (-0.04, 0.61)	0.28 (-0.05, 0.61)	0.25 (-0.07, 0.58)
**SOS**				
Regular cycle	Ref	Ref	Ref	Ref
Irregular cycle	**0.38 (0.05, 0.71)**	**0.36 (0.03, 0.68)**	0.31 (-0.02, 0.64)	0.30 (-0.03, 0.63)
**QUI**				
Regular cycle	Ref	Ref	Ref	Ref
Irregular cycle	**0.38 (0.05, 0.71)**	**0.36 (0.03, 0.68)**	0.32 (-0.01, 0.65)	0.31 (-0.02, 0.63)

BUA = broadband ultrasound attenuation. SOS = speed of sound. QUI = quantitative ultrasound index. SHBG = sex hormone-binding globulin. FAI = free androgen index. QUS measures were log transformed before converted into standardized values. β = standardised beta coefficients. *Adjusted model controlled for age and BMI. Bolder denotes significant association (P < 0.05).

## Discussion

In this cross-sectional study of a population-based sample of premenopausal women (37% overweight or obese), an irregular menstrual cycle was associated with higher quantitative ultrasound measurements including BUA, SOS and QUI. This association was independent of age and BMI but was moderately reduced when further adjusted for hormonal factors, particularly for SHBG and FAI, suggesting that these hormonal factors partially mediate the associations.

Studies have reported a negative association between oligo/amenorrhea and bone mineral density [8-11,19] in female athletes with low BMI. The bone loss was attributed to low levels of estrogen and/or chronic energy deficiency caused by restricted food intake and extensive training [20]. However, these conditions are not common in the general community and these results may not be applicable to young women affected by today's obesity epidemic. In fact, in this population-based sample of young women, the mean BMI was 24.8 kg/m^2 ^which is close to the lower cut-point of overweight (25-30 kg/m^2^) based on the WHO categorization [21]. We found that irregular cycles were associated with higher bone mass and this was partially mediated by SHBG and FAI. This is consistent with other studies' findings that bone mass was associated with higher levels of androgens and lower levels of SHBG [22-24]. Furthermore we found a significant dose-response relationship between hormonal factors and bone mass, especially for FAI.

Few studies have examined bone mass and the association with menstrual irregularity and androgens in population-based samples of premenopausal women though positive associations between amenorrhea and bone mass were reported in women with hirsutism and PCOS - conditions with excess androgen. Endogenous androgens were associated with menstrual irregularity in population-based [12,13] and clinical samples [25], and bone mass in premenopausal women [22,23,26], but these associations were investigated separately. An important strength of our study, therefore, was its population-based sample and ability to examine the role of hormonal factors.

The mechanism for the association between menstrual irregularity, hormonal factors and bone mass is not clear. However the association between menstrual irregularity and bone mass had a moderate reduction in strength and lost significance when further adjusted for either SHBG or FAI separately. This suggests that bone mass could be influenced by SHBG through the regulation of free testosterone levels. Our result is consistent with a prospective study which examined 231 women aged 32-77 years over 2-8 years period and found, in addition to age and weight, SHBG was a strong independent predictor of BMD[26]. A recent editorial discussed a potential dual role for SHBG (inhibitor or facilitator) in regulating sex hormone action [27] following a genetic study of polymorphisms in the SHBG gene promoter which found that serum levels of SHBG were positively associated with BMD in elderly men [28]. However our result favours the free hormone hypothesis that SHBG inhibits sex hormone bioavailability, rather than augments sex hormone action.

There were several limitations which should be considered when interpreting these findings. First, menstrual cycle characteristics were obtained by self-administered questionnaire which did not include specific criteria for women to assess their cycle regularity. This may have resulted in misclassification. However, it is unlikely that this error would be differential with respect to bone mass or hormonal factors since both QUS and hormonal factors were measured at the same time that women reported their menstrual cycles. Therefore the association between irregular cycles and bone mass may be underestimated in our study. Second, we did not have dual-energy x-ray absorptiometry (DXA) measurement of BMD; however QUS results correlate well with BMD measured by DXA at the heel [29] and predict fracture risk similarly to DXA [30]. This study was conducted in community centres across Australia so DXA measurements were not feasible. Furthermore, use of a portable QUS machine avoided standardisation issues which could have been a major problem had we used multiple DXA instruments at different sites. Third, we did not assess endogenous estrogens which show substantial variation during the menstrual cycle. Clearly, estrogens play a major role in regulating bone mass in both males and females. Previous studies examining both estrogens and androgens in premenopausal women found that androgen but not estrogen was associated with bone mass [22,26] suggesting premenopausal estrogen levels may be sufficient in most women. Due to logistic issues, the blood sample collection was not timed with the menstrual cycle possibly introducing some measurement error as testosterone varies with the menstrual cycle [31]. Another limitation is that some young women with irregular cycles may have had undiagnosed PCOS. However, this sample of young women had testosterone levels typically within the normal range. Exclusion of eight women who had testosterone levels higher than 2.9 nmol/l (the upper limit of the laboratory's reference range for premenopausal women with normal menstrual cycles and no evidence of polycystic ovaries on ultrasound) or 24 women who had SHBG levels less than 24 nmol/l (the lowest levels for follicular phase for premenopausal women) made little difference to the findings. In addition, adjustment for a history of ever being told by a doctor they had PCO or PCOS did not decrease the associations. Further, the relatively low percentage of eligible participants with complete data may limit the generalizability of our findings to the general population. Finally, we cannot be certain of the causal direction of the associations observed due to the cross-sectional design of this study. Thus, a longitudinal study is necessary to confirm these findings.

## Conclusions

Irregular menstrual cycles were associated with higher bone mass in this population-based sample of premenopausal women suggesting menstrual disturbance should continue to be evaluated but may be less harmful for bone mass than previously believed. The association between menstrual irregularity and bone mass was partially mediated by markers of androgen status especially free testosterone.

## Competing interests

The authors declare that they have no competing interests.

## Authors' contributions

SW contributed to the design of the study, performed the statistical analysis and drafted the manuscript. AV, GJ and TD contributed to the study design, data collection and revised the manuscript critically. RT and PO participated in analysis and interpretation of data. All authors read and approved the final manuscript.

## Pre-publication history

The pre-publication history for this paper can be accessed here:

http://www.biomedcentral.com/1471-2474/11/288/prepub
